# Analgesia and Fascia Iliaca Compartment Block Utilisation for Neck-of-Femur Fracture Patients: A Closed-Loop Audit

**DOI:** 10.7759/cureus.97771

**Published:** 2025-11-25

**Authors:** Talha Ahmed, Hassan Imtiaz, Rabia Asghar, Muhammad Zaeem, Rizwana Monzur, Atizaz A Jan, Omran Alkhatib

**Affiliations:** 1 Trauma and Orthopaedics, Poole Hospital, University Hospitals Dorset NHS Foundation Trust, Poole, GBR; 2 Endocrinology and Diabetes, Royal Bournemouth Hospital, University Hospitals Dorset NHS Foundation Trust, Bournemouth, GBR; 3 Anesthesia and Critical Care, District Headquarters Hospital, Faisalabad, PAK

**Keywords:** emergency department(ed), fascia iliaca compartment block, hip analgesia, neck of femur fracture (nof), quality improvement (qi)

## Abstract

Introduction

Neck-of-femur (NOF) fractures are a major source of morbidity and mortality among older adults. Timely and effective pain management, including fascia iliaca compartment block (FICB), is recommended to optimise outcomes and minimise opioid-related complications. This study compares two audit cycles assessing improvements in analgesic practice, FICB utilisation, and adherence to national guidelines following targeted educational and procedural interventions.

Methods

Two retrospective audit cycles were conducted in the emergency department at a district general hospital. Cycle 1 (September-October 2023, n = 40) and Cycle 2 (November-December 2024, n = 40). Audit standards were derived from NICE CG124 and RCEM guidance. Following the first cycle results, a mandatory checklist was introduced and disseminated amongst relevant team members, aimed at improving clinical practice. Measured outcomes included time to first analgesia, time to FICB, proportion receiving FICB, pain re-evaluation documentation, and complications. Continuous data were compared using independent samples t-tests; categorical data were analysed using chi-square tests (p < 0.05).

Results

FICB provision remained similar (Cycle 1: 65.0%, n=26 vs Cycle 2: 65%, n=26, p = 0.74), but both time to first analgesia and time to FICB improved significantly, from 130 minutes to 38.8 minutes (p < 0.001) and from 228 minutes to 63.4 minutes (p = 0.002), respectively. Documentation of pain re-evaluation showed no significant change (52.5%, n=21 versus 50.0%, n=20, p = 0.99). No FICB-related complications were reported.

Conclusion

Simple interventions such as the use of a mandatory checklist can significantly improve the timing of first analgesia and FICB administration. However, it did not lead to an increase in block utilisation or documentation of pain reassessment. Further quality improvement projects should focus on system-level strategies to embed pain management protocols for neck of femur fracture patients presenting to the emergency department.

## Introduction

Hip fractures represent one of the most common and serious injuries affecting older adults, with an estimated incidence of 957 cases per 100,000 people, and a projected rise in global incidence due to population ageing [1]. A large study reported a 30-day mortality rate after hip fracture of approximately 9-10% and a one-year mortality rate approaching 33-35% in older cohorts [2,3]. Persistent pain after hip fracture is common and contributes to poor functional recovery [4]. Untreated or undertreated pain also increases the risk of delirium and is associated with prolonged length of stay and worse outcomes [5]. 

Systemic opioids are frequently used but are associated with adverse effects in frail older people, including respiratory depression, sedation, and constipation; these adverse effects can impede rehabilitation and increase delirium risk [6]. Consequently, national guidance emphasises early pain assessment, timely analgesia, and consideration of regional nerve blocks. NICE Clinical Guideline CG124 [7] recommends immediate pain assessment at presentation and consideration of nerve blocks (including FICB) for hip fracture patients where appropriate. RCEM guidance [8] similarly specifies early pain scores (assessment within 15 minutes) and re-evaluation within 30 minutes after analgesia, and it advocates nerve block use when clinical skill and resources permit.

Fascia iliaca compartment block (FICB) provides effective regional analgesia by targeting the femoral, lateral femoral cutaneous, and obturator nerve territories. Landmark and ultrasound-guided techniques have been widely studied; landmark approaches are simple and popular in emergency settings, while ultrasound guidance increases accuracy and reduces failure rates [9-12]. Randomised controlled trials and systematic reviews report that FICB improves pain scores, reduces opioid consumption, and may reduce delirium and opioid-related complications in hip-fracture patients [13-15]. Despite this evidence, national audits and single-centre studies report variable uptake of FICB in emergency departments, typically between 45-70%, with frequent delays to the first analgesic and to block administration [16-18].

We conducted a closed-loop audit to evaluate whether structured education and a mandatory analgesia checklist improved adherence to NICE and RCEM pain management standards for neck-of-femur fracture patients.

## Materials and methods

A quality improvement project was conducted in the Emergency Department (ED) at Macclesfield District General Hospital, a district general hospital in the United Kingdom. Cycle 1 (September-October 2023) served as the baseline, while Cycle 2 (November-December 2024) evaluated the impact of implemented interventions. Audit standards were derived from NICE CG124 guidelines [7] and RCEM guidelines for pain management in neck of femur fracture patients [8], and are presented in Table 1.

**Table 1:** Audit Standards

Audit Standard	Target Compliance (%)
FICB performed where applicable	100%
If FICB not performed, were contraindications documented	100%
Pain re-evaluation within 30 min of analgesia administration	100%

Targeted interventions between audit cycles included organisations of practical sessions to familiarise ED clinicians with the procedure for administering FICB. Also, the implementation of a mandatory checklist (Appendix) for analgesia administration amongst all patients presenting to the emergency department with a neck of femur fracture was undertaken. This checklist included patient demographics (weight, height), pain assessment at arrival, 30 and 60 mins, with separate boxes for analgesia administered at each time. It also included a box for FICB administration, with time and choice of anaesthetic used, name of clinician administering it, and observations and pain score assessment before and after the procedure (at 5 and 10 mins). In cases where FICB was not administered, a separate line was provided to document the rationale. 

Patients aged ≥18 years presenting with radiologically confirmed NOF fractures were included. Delayed presentations, inter-hospital transfers, inpatient falls causing NOF fractures, or incomplete records met the exclusion criteria.

Measured outcomes involved documentation of the time to first analgesia (from triage to administration), time to FICB (from triage to procedure), percentage of patients with suspected NOF fractures receiving FICB, proportion without FICB and no contraindications, pain re-evaluation within 30 minutes of initial analgesia, and any FICB-related complications.

Data sources utilised included electronic medical records and emergency department clerking proformas. This was stored on the trust's intranet on a Microsoft Excel spreadsheet (Microsoft Corp., Redmond, WA) and analysed on JASP software v. 0.18.3 (University of Amsterdam, Amsterdam, the Netherlands). Continuous variables were compared using independent-sample t-tests, while Categorical data were compared using chi-square tests. Statistical significance was set at p < 0.05. This Quality Improvement project was conducted under clinical governance registration and did not require ethical approval.

## Results

A total of 80 patients were included across both cycles (Cycle 1: n = 40; Cycle 2: n = 40). FICB was performed in 26 of 40 (65.0%) patients amongst both cycles, showing no significant change (p = 0.74). Similarly, 14 patients (35%) did not receive FICB for both cycles; amongst these, none (0%) had any contraindications documented for not administering FICB during the first cycle, while 9/14 (64.3%) had contraindications mentioned for those evaluated in the second cycle. Documentation of pain re-evaluation within 30 minutes was comparable (52.5% vs 50.0%, p = 0.99). A summary of results is presented in Table 2. 

**Table 2:** Summary Results for Cycles 1 and 2 FICB: fascia iliaca compartment block; SD: standard deviation

Parameter	Cycle 1 (Sept–Oct 2023)	Cycle 2 (Nov–Dec 2024)
Sample size	n=40	n=40
FICB performed (n, %)	26 (65%)	26 (65%)
No FICB (n, %)	14 (35%)	14 (35%)
Contraindications documented amongst ‘No FICB’ group (n/N, %)	0/14 (0%)	9/14 (64.3%)
Pain re-evaluation within 30 min (n, %)	21 (52.5%)	20 (50%)
Mean time to first analgesia in minutes (mean ± SD)	130 ± 28.8	38.8 ± 13.2
Mean time to FICB in minutes (mean ± SD)	228 ± 36.2	63.4 ± 19.1
FICB complications	None	None

Time to first analgesia and time to FICB both improved markedly in Cycle 2. The mean time to first analgesia decreased from an average of 130 ±28.8 minutes in Cycle 1 to 38.8 minutes ± 13.2 in Cycle 2 (p < 0.001), while the mean time to FICB decreased from 228 ± 36.2 minutes to 63.4 ± 19.1 minutes (p = 0.002). No FICB-related complications were reported in either cycle. Comparison between the cycles is presented in Table 3. 

**Table 3:** Statistical Comparison Between Cycles *chi-square test **Independent samples t-test FICB: fascia iliaca compartment block

Outcome	Statistical test	p-value	Interpretation
Pain re-evaluation	χ² = 0.00*	0.99	No significant change
Time to first analgesia	t(21) = −12.01**	<0.001	Significantly shorter in Cycle 2
Time to FICB	t(8) = −4.41	0.002	Significantly shorter in Cycle 2

## Discussion

The results of this quality improvement project highlight that targeted educational and procedural interventions can markedly improve the timeliness of analgesic delivery in hip fracture management. The introduction of structured education and an analgesia proforma resulted in the mean time to first analgesia being reduced from 130 minutes to 38.8 minutes, and the mean time to FICB from 228 minutes to 63.4 minutes, both representing statistically significant improvements (p < 0.001 and p = 0.002, respectively). By introducing a structured checklist and delivering focused training sessions, this project achieved a threefold improvement in analgesic response time, aligning with the National Hip Fracture Database (NHFD) recommendations for early pain assessment and timely analgesia [16].

Despite these advances, FICB utilisation remained unchanged at 65%, comparable to the 45-70% range reported in other UK and international studies [16-18]. The lack of improvement in block provision may reflect systemic and behavioural barriers identified in prior audits, such as inconsistent staff training, variable confidence among junior clinicians, and time constraints during peak department activity [17,18]. Nevertheless, documentation quality improved substantially, with contraindications recorded in 64.3% of non-FICB cases post-intervention, compared to none in the baseline cycle. This suggests that the checklist effectively enhanced clinical accountability and awareness of documentation standards, even if it did not increase block frequency.

The observed improvements mirror the findings of Foss et al. [10] and Steenberg and Møller [13], who reported that FICB training initiatives significantly reduced delays in analgesia administration, though sustained adherence required continuous institutional support. Randomised controlled trials have further demonstrated that FICB provides superior analgesia compared with systemic opioids, resulting in reduced pain scores, decreased opioid consumption, and lower incidence of delirium and postoperative complications [11,13-15]. For example, Abou-Setta et al. [12] reported that patients receiving FICB experienced an average 35-50% reduction in opioid use compared to those treated with systemic analgesia alone, while Wan et al. [15] found significant reductions in postoperative delirium and hospital length of stay. These benefits underline the importance of embedding regional anaesthetic techniques into emergency department pathways for frail older adults with hip fractures.

Importantly, the absence of FICB-related complications in both audit cycles aligns with the established safety profile reported across multiple studies [11,15]. Dolan et al. [19] further demonstrated that ultrasound-guided approaches significantly reduce procedural failures and inadvertent vascular puncture compared to traditional landmark techniques, supporting wider adoption where resources permit. This reinforces that concerns about procedural safety should not act as a deterrent to block implementation, particularly when structured training and supervision are provided.

Nevertheless, barriers to widespread FICB adoption persist. Okereke et al. [17] and Evans et al. [20] both highlighted that inconsistent training provision, high staff turnover, and uncertainty surrounding contraindications remain leading causes of underutilisation. These findings are consistent with the current audit, where more than one-third of patients eligible for FICB did not receive one, suggesting that staff familiarity and resource availability continue to influence practice variability. Additionally, time pressures in emergency departments, especially during high-volume periods, may discourage clinicians from performing technically demanding procedures such as nerve blocks despite their proven benefits.

This study has several limitations. As a retrospective evaluation, it depended on the accuracy and completeness of clinical documentation. Unmeasured confounders, such as staffing patterns or patient volume in the emergency department, may have influenced results. Third, the sample size, while representative of a single centre, limits generalisability and warrants a multi-centre approach to aim for higher numbers. The absence of long-term follow-up to assess the sustainability of improvement beyond the second audit cycle and the limited assessment of staff-level factors (e.g., training uptake, confidence, or resource constraints) that may explain the unchanged FICB utilisation rate serve as potential limitations. The potential for data entry or documentation bias, as accuracy depended on electronic records and checklist completion, may also limit the study. Finally, outcome measures were process-based rather than patient-centred; future work should assess pain scores, opioid use, and functional recovery post-intervention.

## Conclusions

This quality improvement project demonstrates that educational interventions and structured documentation improved the timeliness of analgesia and FICB delivery for hip-fracture patients in the emergency department. However, overall FICB utilisation and pain reassessment documentation remain suboptimal. Continued progress requires system-level strategies, integrating electronic prompts, dedicated FICB champions, and ongoing training to achieve consistent, guideline-compliant analgesia for all eligible patients.

## Figures and Tables

**Figure 1:**
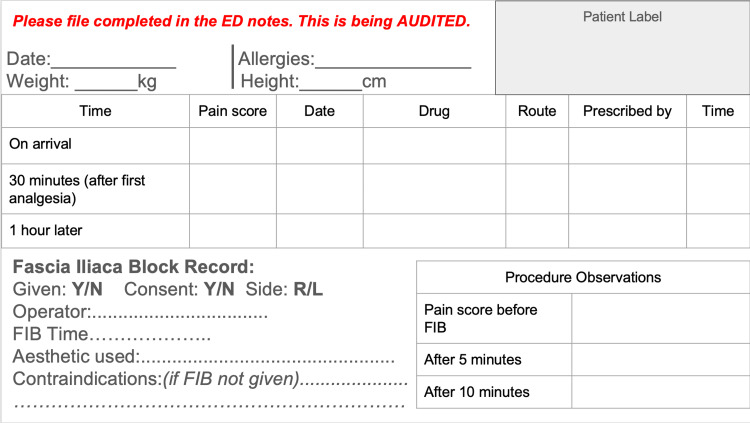
Appendix 1 (Anaglesia adminsitration checklist, mandatory for all suspected neck of femur fractures)

## Appendices

Figure 1 shows the anaglesia administration checklist, mandatory for all suspected neck of femur fractures.
